# Modulators of Caregiver Distress and Corresponding Impacts on Persons With Dementia

**DOI:** 10.1093/geroni/igab046.499

**Published:** 2021-12-17

**Authors:** Michaella Trites, Sebastian Santana, Debra Sheets, Andre Smith, Robert Stawski, Stuart MacDonald

**Affiliations:** 1 University of Victoria, Victoria, British Columbia, Canada; 2 Oregon State University, Corvallis, Oregon, United States

## Abstract

Extensive literature documents the detrimental effects of caregiver distress (CD) for caregivers. Less is known about the impact that CD exerts upon their care recipients, particularly persons with dementia (PwD). Using multilevel modeling, this study employed dyadic data from the Voices in Motion study to examine time-varying within-person associations between key caregiver and care recipient indicators of psychosocial function. An initial dyadic coupling model indicated that cognitive functioning for PwD and caregiver well-being significantly predicted shifts in CD. A second time-varying dyadic model found that, within dyads, high levels of CD predicted lower positive affect and increased depression scores in PwD. Most sociocognitive interventions target a sole member of a dyad; our results suggest that both dyad members are impacted simultaneously. Caregiver well-being impacts CD, which in turn, impacts well-being in PwD. The symbiotic relationship between care partners and PwD is nuanced, with further research required to understand the interdependencies.

